# Mental health and sexual health - two modern definitions and their impact on holistic care

**DOI:** 10.1192/j.eurpsy.2021.1196

**Published:** 2021-08-13

**Authors:** W. Kosmowski

**Affiliations:** Department Of Psychiatry, Collegium Medicum, Nicolaus Copernicus University, Bydgoszcz, Poland

**Keywords:** mental health, sexual health, philosophy of medicine

## Abstract

**Introduction:**

Definitions of health in different branches of medicine are one of the key paradigms in medical sciences. Nowadays, there are two distinct definitions of sexual health and mental health. The definition of sexual health, as well as sexual rights, was proposed by the World Health Organization (WHO, 2006), and the definition of mental health was published in World Psychiatry (Galderisi et al, 2015).

**Objectives:**

The analysis and comparison of these two definitions: mental health and sexual health are two main objectives of this study.

**Methods:**

The analysis was carried out in three areas: logic, philosophical aspects (values) and the impact of other disciplines.

**Results:**

The definition of sexual health reveals a eudaimonistic approach, whereas the definition of mental health is based on a holistic paradigm. Regarding the main principles in the definition of sexual heath, one can identify the following values: well-being, pleasure, safety, sexual rights – compared to harmony, empathy, coping skills, universal values in the definition of mental health. Sexual rights are a constitutive part of sexual health. There is no comparative element in the definition of mental health (e.g. the rights of mentally disabled persons).

**Conclusions:**

These two definitions can have different effects on the prophylaxis and therapy of patients. It all depends on the specific context of care (sexology or psychiatry). Sometimes universal values matter and sometimes not. This is contradictory. Consistency is needed between definitions and practices.

